# Evaluation of System-Level, Passive Chlorination in Gravity-Fed Piped Water Systems in Rural Nepal

**DOI:** 10.1021/acs.est.2c03133

**Published:** 2022-09-20

**Authors:** Yoshika S. Crider, Sanjeena Sainju, Rubika Shrestha, Guillaume Clair-Caliot, Ariane Schertenleib, Bal Mukunda Kunwar, Madan R. Bhatta, Sara J. Marks, Isha Ray

**Affiliations:** † Energy & Resources Group, University of California, Berkeley, Berkeley, California 94305, United States; ‡ Division of Epidemiology and Biostatistics, University of California, Berkeley, Berkeley, California 94305, United States; § Department of Environmental Science and Engineering, Kathmandu University, Dhulikhel 45200, Nepal; ∥ Helvetas Nepal, Lalitpur 44700, Nepal; ⊥ Eawag, Swiss Federal Institute of Aquatic Science and Technology, Duebendorf 8600, Switzerland

**Keywords:** chlorine, safe drinking water, rural water supply, passive chlorination

## Abstract

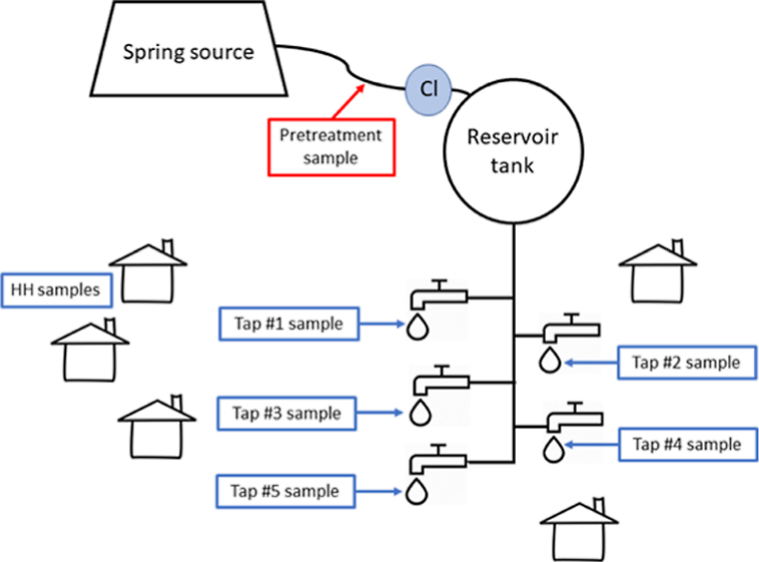

Over 2 billion people globally lack access to safely managed drinking water. In contrast to the household-level, manually implemented treatment products that have been the dominant strategy for gaining low-cost access to safe drinking water, passive chlorination technologies have the potential to treat water and reduce reliance on individual behavior change. However, few studies exist that evaluate the performance and costs of these technologies over time, especially in small, rural systems. We conducted a nonrandomized evaluation of two passive chlorination technologies for system-level water treatment in six gravity-fed, piped water systems in small communities in the hilly region of western Nepal. We monitored water quality indicators upstream of the treatment, at shared taps, and at households, as well as user acceptability and maintenance costs, over 1 year. At baseline, over 80% of tap samples were contaminated with *Escherichia coli*. After 1 year of system-level chlorination, only 7% of those same taps had *E. coli*. However, 29% of household stored water was positive for *E. coli*. Per cubic meter of treated water, the cost of chlorine was 0.06–0.09 USD, similar to the cost of monitoring technology installations. Safe storage, service delivery models, and reliable supply chains are required, but passive chlorination technologies have the potential to radically improve how rural households gain access to safely managed water.

## Abstract 2

Few studies have evaluated the performance of passive chlorination technologies in small, rural water systems. This study reports results from a yearlong passive chlorination implementation and finds that these technologies can significantly increase community-wide access to safe drinking water.

## Introduction

Access to safe drinking water is a human right and a public health priority,^1^ yet over 2 billion people globally lack access to clean, affordable, and reliably supplied water.^2^ This contributes to a high global burden of diarrheal disease, which is estimated to be the eighth leading cause of death around the world.^3^ While country-level measures of access show improving trends overall, these data mask within-country spatial inequalities. Rural areas lag behind urban areas in water access across all regions of the world.^4^

Household, or point-of-use (POU), drinking water treatment has been the dominant strategy for ensuring safe drinking water where effective, centralized treatment systems do not exist.^5−7^ POU treatments, such as household filters, solar disinfection, boiling, or manually adding chlorine products, require daily behavior change and place the responsibility for treatment on individuals within households; these individuals tend to be women and girls, who are most often tasked with household water management.^8^ Modeling studies have concluded that near-perfect levels of correct and consistent use of POU water treatment are required to realize their health benefits, yet lower use is typically observed in real world trials of POU interventions.^9,10^

In low-resource settings where piped water infrastructure exists but centralized treatment is inadequate, passive in-line chlorination technologies that require no electricity are being implemented as a potentially more effective alternative to POU options, and several new technologies have been developed and tested in recent years.^11^ Because of their limited treatment capacity compared to large-scale centralized treatment infrastructure, these technologies are typically most appropriate at a decentralized scale in a distribution network, for example, a small neighborhood or apartment building. However, some technologies may be suitable as a fully centralized treatment option for small, rural village water supplies. The relative simplicity of these technologies may be especially appropriate for such settings since size and resources limit the operation and maintenance of full-scale water treatment facilities. However, while several passive chlorination technologies are compatible with rural piped water system infrastructure, few studies to date have evaluated the technical performance of these technologies in small rural piped systems,^12,13^ and there exist almost no data on long-term costs of operation and maintenance.^14^

Our objective was to evaluate the impact of centralized, system-level implementation of these technologies on system and household water quality and user acceptability as well as the associated costs for operation and maintenance over 1 year. Our study was nested within a larger rural water safety intervention (REACH-Nepal), which implemented and evaluated a combination of water safety planning (WSP) interventions. In Nepal, WSPs are widely promoted strategies to reduce water system risks by identifying local hazards, implementing multi-barrier control measures, and following a regular monitoring plan. Within REACH-Nepal, we conducted a nonrandomized evaluation of two tablet-based, passive chlorination technologies for system-level water treatment in six gravity-fed, piped water systems in small communities in Karnali Province, located in the hilly region of western Nepal. Approximately half of the rural population in Nepal is estimated to have access to piped water,^15^ but quality is poor. A prior assessment of microbial water quality during October through December in communities of this region found that 68% of water sources and 81% of household stored water samples had fecal contamination.^16^ This may be an underestimate of annual peak contamination trends because fecal contamination is often higher during wet seasons, typically June through September in Nepal.^17^ However, according to the 2016 Nepal Demographic and Health Survey, only 12% of rural households treat water prior to drinking.^15^ In this setting, an effective passive chlorination technology for piped water systems would have high potential to reduce exposure to fecal contamination through drinking water.

## Methods

### Study Setting and Design

The REACH-Nepal parent study was a collaboration between researchers at the Swiss Federal Institute of Aquatic Science and Technology (Eawag) and the international NGO Helvetas-Nepal working in 33 rural communities. Full details of the parent trial are described elsewhere.^18^ In summary, the intervention included construction of field laboratories, water system upgrades, and water quality monitoring with centralized data management. Local NGO workers were trained to manually chlorinate the gravity-fed piped water supply at reservoir tanks in four treatment communities, and bleaching powder was provided for free. However, no enrolled communities consistently practiced system-level manual chlorination. In this sub-study, we evaluated two chlorination technologies that could be installed at the system level to automatically chlorinate the piped water supply. We selected two adjacent communities from the pool of 21 treatment communities, each of which had one or more piped water systems, enrolled in the parent study. These two communities were selected because they had six water distribution systems all within a half day’s walking distance, which made repeated sampling and monitoring of multiple installations logistically feasible by a small field team. Each water distribution system had a similar design, including a spring source, a 2.5–5 m^3^ concrete reservoir tank, and a gravity-fed piped distribution system to outdoor taps. Systems ranged in size from 6 to 16 taps serving 16–28 households, with 1–6 households per tap, according to system planning documents. Two reservoir tanks shared the same spring source; the remaining four had separate spring sources. Each community had a water users’ committee to manage the water supply. The small system repairs were done by community members designated as “village maintenance workers”.

### Passive Chlorination Technologies

**Figure 1:**
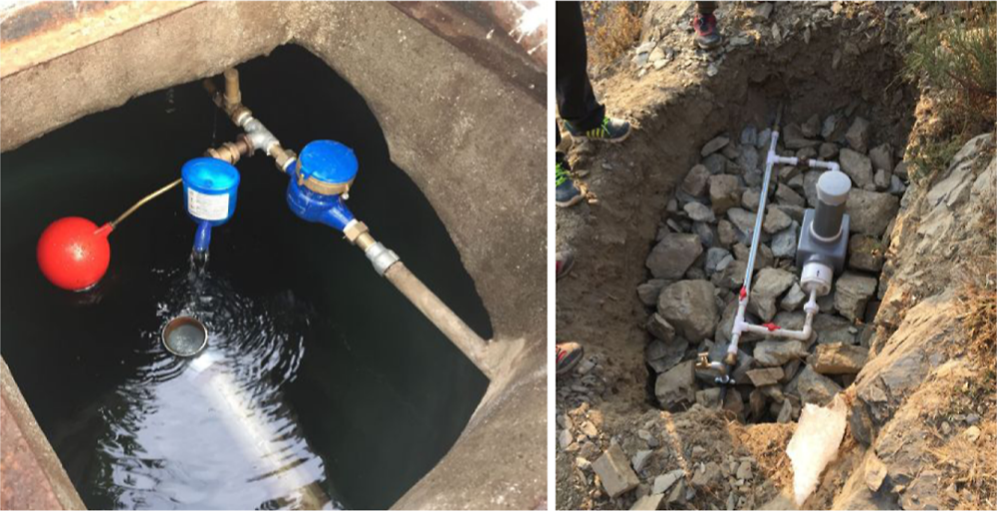
(Left) Aquatabs Flo technology installed at the inlet to a reservoir tank. Pretreatment samples were collected from the unchlorinated bypass. (Right) PurAll 100 technology installed in-line just upstream of a reservoir tank. Pretreatment samples were collected from a sampling tap, visible just upstream of the device.

Technologies were installed upstream of system reservoir tanks. We selected passive chlorination technologies based on their compatibility with existing infrastructure and their availability in Nepal (imported by local distributors located in Kathmandu and Pokhara); we purchased all chlorinators and refills at the local market price. We hypothesized that they would be similar in terms of disinfection efficacy, with similar chlorine tablet erosion mechanisms but that they would have different costs and labor time required for maintenance, which would affect the feasibility of each option for wider implementation in similar communities. The first technology is marketed as the Aquatabs Flo (Medentech, Wexford, Ireland). It is an “end-line” erosion chlorinator that consists of a small cartridge, filled with solid tablets of trichloro-*s*-triazinetrione (also known as trichlor), that is twisted onto an accompanying adapter at the outflow of a pipe (Figure 1). As water moves through the cartridge, it slowly dissolves and mixes with the tablets through slots in the cartridge channel. There are two ways to adjust dosing. First, increased mixing, and higher dosing, can be achieved by lowering a plastic screw to partially block the channel. Second, upstream of the device, the pipe can be split into two branches to adjust the proportion of water that flows through versus bypassing the cartridge. The advertised cartridge capacity is 180 m^3^ dosed with 1 mg/L chlorine. The device is refilled by swapping out the entire cartridge. This technology has previously been evaluated at household water points in urban Bangladesh,^11,19^ healthcare facilities in Tanzania,^20^ and kiosks in Uganda.^14,21^

The second technology is marketed as PurAll 100 (Easol Ltd., Maharashtra, India). It is an “in-line” T-shaped erosion chlorinator that consists of a rectangular box with a vertical tube containing a cartridge stacked with trichlor tablets, and it is installed in the pipeline (Figure 1). As water moves through the box, it slowly dissolves and mixes with the chlorine tablets through slots at the bottom of the cartridge tube. As tablets dissolve, new tablets drop down in the tube. To adjust the dosing, the pipe is split into two branches upstream of the chlorinator and valves are used to change the proportion of water through the device or bypass. The advertised cartridge capacity is 2500 m^3^ dosed with 1 mg/L chlorine. The device is refilled by unscrewing the top of the tube and swapping out the entire cartridge nested inside. No prior published evaluations of this technology were identified, but it is a similar design to T-shaped erosion chlorinators evaluated elsewhere.^12−14^

Technologies were purposively assigned based on system size, with the higher-capacity PurAll 100 chlorinator assigned to the larger three systems, which we refer to as systems 1B, 2B, and 3B. We refer to smaller three systems assigned to the Aquatabs Flo as systems 1A, 2A, and 3A. During initial site visits, we asked a few community members about prior chlorine experience and provided chlorinated water samples to assess taste and smell acceptability. The responses suggested similar taste and smell acceptability found in other settings.^22^ Thus, to avoid households’ rejection of the chlorinators due to taste and/or smell of chlorine, we initially adjusted dosing to target 1 mg/L at the tap.

### Data Collection and Outcomes

#### Household Water Quality and User Acceptability

**Figure 2:**
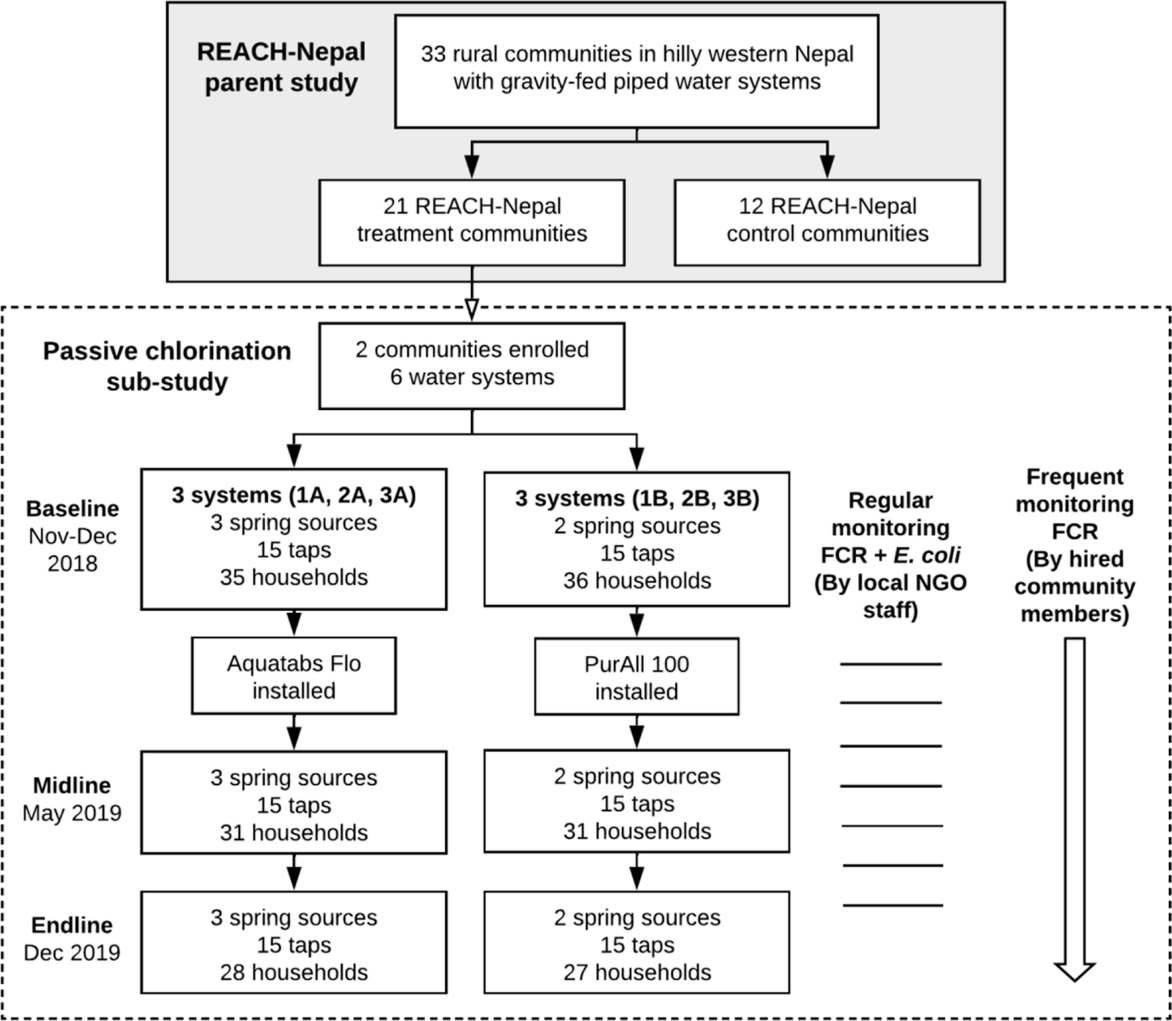
Study flow chart. We define a system as a reservoir tank and its associated piped distribution system with shared taps accessed by households.

We conducted three rounds (Figure 2) of household surveys to assess pre- and post-installation user acceptability, chlorination impacts on household water quality, and water management practices that could influence quality. We collected baseline data from November–December 2018, midline data in May 2019, and endline data in December 2019. At each round, we sampled from household stored drinking water containers and conducted interviews that included questions on the household’s water access, water treatment and storage practices, and perceptions of water quality and safety. We identified households from water system planning documents that listed participating households, then ordered them using Microsoft Excel’s random number generator, and approached them in that order. One adult who made decisions about water management was enrolled in each household until 15 households per system had been enrolled or until all available households had been approached, whichever occurred first. On subsequent visits to each household, we attempted to interview the same individual; if this individual was unavailable, we obtained consent from and interviewed another eligible adult with water management responsibilities. Surveys were conducted in Nepali by native Nepali speakers using tablets with Open Data Kit (ODK) open-source mobile survey software (opendatakit.org).

#### System Water Quality and Sampling Strategy

To evaluate the effectiveness of each technology to improve water quality, we closely monitored free chlorine residual (FCR), *Escherichia coli*, and total coliforms in the distribution systems. We collected pretreatment samples either at a non-chlorinated bypass pipe at the reservoir tank inlet (Aquatabs Flo) or from a sampling tap installed just upstream from the chlorinator (PurAll 100). At each system’s survey round or monitoring visit, one pretreatment sample was collected. At baseline, we randomly selected five taps across each system, downstream of the reservoir tank. Each was assigned a unique tap ID and sampled during the three survey rounds. We conducted additional monitoring visits, separate from survey rounds, from February to November 2019 to ensure that systems were operating correctly. Over seven monitoring visits to each system, trained NGO staff collected and processed a pretreatment sample, samples from the closest and farthest taps from the chlorinator, and a stored drinking water sample from one household near one of the two selected tap locations. The results were recorded on tablets using ODK. Additionally, from December 2018 to November 2019, two community members were hired and trained to measure FCR 1–2 times per week at one tap at each system to flag any non-dosing events.

#### Cost of Operation and Maintenance

We documented the person-hours required for NGO staff and hired community members to monitor and maintain devices during this study. We kept a record of all chlorine refills, starting when chlorination began in late December 2018/early January 2019 until the last refills were recorded in October/November 2019, before endline data collection in December 2019. We installed locally purchased mechanical flow meters at the inlet of each reservoir tank to track the total volume (m^3^) treated per technology installation.

### Sample Collection and Microbial Testing

For tap samples, we turned on taps for 30 s prior to collecting each sample. Household water samples were collected directly from drinking water storage containers. We measured free and total chlorine at the sampling location. For regular FCR monitoring by trained community members, some measurements were taken using a Lovibond low-range pool tester, which has a range of 0.1–3 mg/L Cl_2_ (Tintometer Inc., Sarasota, FL). All other free and total chlorine measurements were collected with a LaMotte DC1500 digital colorimeter and DPD tablets (LaMotte Co., Chestertown, PA), with a range of 0.03–4 mg/L. Samples for *E. coli* and total coliforms were collected in 100 mL Whirl-Pak Thio-bags (Nasco, Fort Atkinson, USA) and filtered through 47 mm diameter, 0.45 μm pore size cellulose filters (MilliporeSigma, Burlington, MA) using a filtration funnel with a manual vacuum pump (DelAgua, UK) and placed on Nissui Compact Dry EC plates (Nissui Pharmaceuticals, Japan) at a mobile field lab. Samples were typically processed within 2 h on-site. Filtration funnels were sterilized with methanol vapor, and sterile water was produced daily by filling a sterilized baby bottle with boiled tap water and sodium thiosulfate to neutralize residual chlorine from the community water supply. Processed samples were transported within 1.5 h on average (range: 0–5 h) in an insulated container to a central field lab installed at the home of a village maintenance worker. There, plates were incubated at 35 ± 2 °C for 24 h in a locally custom-built, solar-powered incubator and *E. coli* and total coliform colonies were counted on each plate. Additional details on equipment construction and methods are described elsewhere.^23,24^ One negative control and one duplicate sample were processed daily for quality assurance and quality control.

### Data Analysis

We cleaned data in STATA version 13 and did analysis in R version 4.0.2. Data and replication scripts are available at https://osf.io/mrtfb/. Colony forming unit (CFU) counts exceeding 300 were above the method limit of detection, and we assigned these a value of 300 for statistical analysis. We assigned a value of 0.5 to 0 counts prior to log transformation. To convert costs to USD, we used a January 1, 2020, exchange rate of 1 USD = 114.34 Nepali rupees (NRS). Differences in outcomes between sample types and between sampling rounds were estimated using linear regression and robust standard errors clustered at the system level using the *estimatr* R package (see the Supporting Information for additional details).

### Ethics

All surveyed households gave verbal informed consent. Prior to enrollment, all households in the selected communities were invited to an outdoor informational meeting where the research team and NGO staff explained the purpose and planned activities of the study; 49 community members attended. The study protocol received ethical approval from the Nepal Health Research Council (Reg. no. 24/2018) as part of ongoing Eawag research activities, from Eawag’s internal ethical review committee (protocol no. 1609_20180227), and from the Committee for the Protection of Human Subjects at the University of California, Berkeley (2018-08-11354).

## Results

We collected data from 71 households at baseline, 62 households at midline, and 55 households at endline. Reasons for loss to follow-up included migration out of the community, attending a funeral or wedding away from the community, and the birth of a baby. We attempted to follow up with missing households but were unable to do so in some cases.

### Community Water Access and Uses

Water supply services were named as a main community concern by 11 and 22% in Aquatabs Flo and PurAll 100 communities, respectively (Supporting Information Table 1). At baseline, all households reported that the piped water supply was their primary drinking water source in both wet and dry seasons. The majority (67/71) of households reported monthly payments for water supply from shared taps ranging from 10 to 20 NRS (0.08–0.17 USD). Across both wet and dry seasons, 59% of respondents reported that they had experienced intermittently supplied water (i.e., <24 h of availability per day), mainly during the dry season. Across all households, the roundtrip water collection time was on average 6.8 min (range: 2–40 min). The majority of households collected water at taps in containers (61%), with the rest of households using either a flexible pipe they pushed onto the tap to pipe water directly into their home (23%) or a combination of the two methods (17%).

### Survey Rounds

At baseline, 60% of pretreatment and 87% of tap samples were positive for *E. coli* (Table 1). The degree of contamination was fairly stable through the distribution system, with an average increase of 0.14 [95% confidence interval (CI): −0.95 to 1.24] log_10_ CFU/100 mL *E. coli* between pretreatment and tap samples. Among household stored water samples at baseline, 77% had *E. coli* present. At midline, 80% of pretreatment samples, 13% of tap samples, and 55% of household stored water samples were positive for *E. coli.* Three of the four contaminated tap samples had FCR > 0.1 mg/L, although all would be prioritized as low risk (1–10 CFU/100 mL *E. coli*) according to World Health Organization guidelines.^25^ There was an average reduction of 0.95 (95% CI: −1.85 to −0.03) log_10_ CFU/100 mL *E. coli* between pretreatment and tap samples. FCR was detectable (>0.1 mg/L) at 73% of taps and 27% of households. At endline, 80% of pretreatment samples, 7% (2/30) of tap samples, and 29% of household stored water samples were positive for *E. coli*. Both contaminated tap samples had FCR >0.1 mg/L and would be considered low and medium risk (11–100 CFU/100 mL *E. coli*). There was an average reduction of 1.15 (95% CI: −2.25 to −0.05) log_10_ CFU/100 mL *E. coli* between pretreatment and tap samples. FCR was detectable at 93% of taps and 49% of households.

**Table 1:** Water Quality Results at Each Survey Round (Baseline, Midline, and Endline)^a^^,^^c^

	Aquatabs Flo			PurAll 100			combined			
	baseline	midline	endline	baseline	midline	endline	baseline	midline	endline	difference: endline vs baseline
**pretreatment (RVT)**	*n* = 3	*n* = 3	*n* = 3	*n* = 2	*n* = 2	*n* = 2	*N* = 5	*N* = 5	*N* = 5	(95% CI)
*E. coli* present (proportion of samples)	0.67 (0.58)	1.00 (0.00)	0.67 (0.58)	0.50 (0.71)	0.50 (0.71)	1.00 (0.00)	0.60 (0.55)	0.80 (0.45)	0.80 (0.45)	0.2 (−0.25, 0.65)
total coliform present (proportion)	1.00 (0.00)	1.00 (0.00)	1.00 (0.00)	1.00 (0.00)	1.00 (0.00)	1.00 (0.00)	1.00 (0.00)	1.00 (0.00)	1.00 (0.00)	0
*E. coli* log_10_ (CFU/100 mL)	0.43 (0.71)	1.17 (0.24)	0.83 (1.20)	0.57 (1.22)	0.09 (0.55)	1.02 (0.77)	0.48 (0.79)	0.74 (0.68)	0.91 (0.94)	0.42 (−0.94, 1.79)
total coliform log_10_ (CFU/100 mL)	2.48 (0.00)	2.48 (0.00)	2.48 (0.00)	2.42 (0.08)	2.29 (0.27)	2.48 (0.00)	2.45 (0.05)	2.40 (0.17)	2.48 (0.00)	0.02 (−0.04, 0.08)
free chlorine >0.1 mg/L (proportion)		0.00 (0.00)	0.00 (0.00)		0.00 (0.00)	0.00 (0.00)		0.00 (0.00)	0.00 (0.00)	
**taps**	*n* = 15	*n* = 15	*n* = 15	*n* = 15	*n* = 15	*n* = 15	*N* = 30	*N* = 30	*N* = 30	
*E. coli* present (proportion)	1.00 (0.00)	0.00 (0.00)	0.13 (0.35)	0.73 (0.46)	0.27 (0.46)	0.00 (0.00)	0.87 (0.35)	0.13 (0.35)	0.07 (0.25)	–0.80 (−1.03, −0.57)^b^
total coliform present (proportion)	1.00 (0.00)	0.27 (0.46)	0.20 (0.41)	1.00 (0.00)	0.33 (0.49)	0.00 (0.00)	1.00 (0.00)	0.30 (0.47)	0.10 (0.31)	–0.90 (−1.08, −0.72)^b^
*E. coli* log_10_ (CFU/100 mL)	1.05 (0.58)	–0.30 (0.00)	–0.19 (0.35)	0.20 (0.49)	–0.10 (0.39)	–0.30 (0.00)	0.63 (0.68)	–0.20 (0.29)	–0.25 (0.25)	–0.87 (−1.43, −0.32)^b^
total coliform log_10_ (CFU/100 mL)	2.40 (0.13)	0.09 (0.83)	–0.06 (0.56)	2.33 (0.16)	0.06 (0.77)	–0.30 (0.00)	2.37 (0.14)	0.07 (0.79)	–0.18 (0.41)	–2.54 (−2.80, −2.29)^b^
free chlorine >0.1 mg/L (proportion)		0.67 (0.49)	1.00 (0.00)		0.80 (0.41)	0.87 (0.35)		0.73 (0.45)	0.93 (0.25)	
FCR (mg/L)		0.50 (0.41)	0.66 (0.44)		0.65 (0.62)	2.46 (1.42)		0.57 (0.52)	1.56 (1.38)	
**households**	*n* = 34	*n* = 31	*n* = 28	*n* = 36	*n* = 31	*n* = 27	*N* = 70	*N* = 62	*N* = 55	
*E. coli* present (proportion)	0.76 (0.43)	0.65 (0.49)	0.29 (0.46)	0.78 (0.42)	0.45 (0.51)	0.30 (0.47)	0.77 (0.42)	0.55 (0.50)	0.29 (0.46)	–0.48 (−0.67, −0.29)^b^
total coliform present (proportion)	0.91 (0.29)	0.90 (0.30)	0.75 (0.44)	0.92 (0.28)	0.71 (0.46)	0.44 (0.51)	0.91 (0.28)	0.81 (0.40)	0.60 (0.49)	–0.31 (−0.65, 0.02)
*E. coli* log_10_ (CFU/100 mL)	1.06 (0.97)	0.42 (0.84)	0.03 (0.75)	0.68 (0.78)	0.43 (0.99)	–0.06 (0.45)	0.86 (0.90)	0.43 (0.91)	–0.02 (0.62)	–0.88 (−1.37, −0.39)^b^
total coliform log_10_ (CFU/100 mL)	1.81 (0.99)	1.74 (0.94)	0.90 (1.13)	1.67 (0.93)	1.04 (1.15)	0.42 (0.99)	1.74 (0.96)	1.39 (1.10)	0.66 (1.08)	–1.07 (−1.73, −0.41)^b^
free chlorine >0.1 mg/L (proportion)		0.23 (0.43)	0.32 (0.48)		0.32 (0.48)	0.67 (0.48)		0.27 (0.45)	0.49 (0.50)	
FCR (mg/L)		0.09 (0.14)	0.11 (0.13)		0.25 (0.60)	1.19 (1.63)		0.17 (0.44)	0.64 (1.26)	

a All values are mean (SD), unless otherwise specified. CFU = colony forming units.

b *p* < 0.01.

c At baseline, we were unable to collect a household stored water sample from one respondent because she was unable to enter her home while she was menstruating. This practice of exclusion, called *chhaupadi*, is becoming less common in rural Nepal.

### FCR and *E. coli* Monitoring

**Figure 3:**
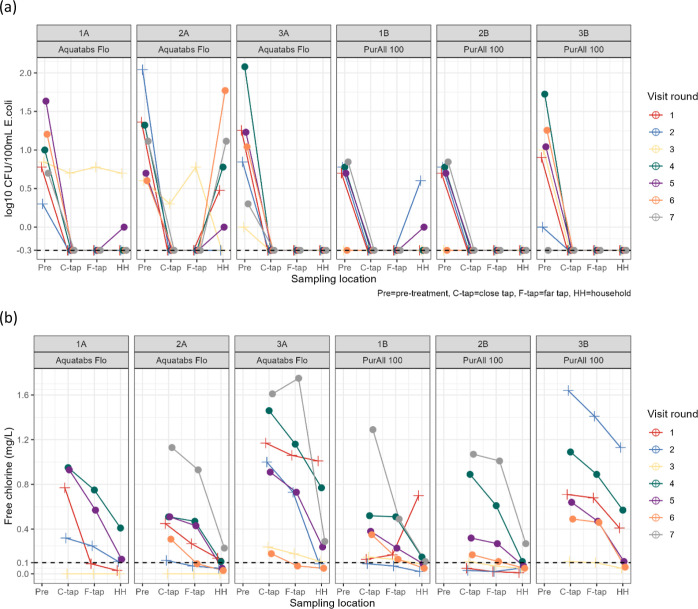
(a) *E. coli* (log CFU/100 mL) across seven monitoring visits from February to November 2020. For each round of sampling, the line connects the specified observed water quality parameter from the pretreatment sampling location, the tap closest to the chlorinator, the tap farthest from the chlorinator, and one household nearby one of the selected taps. Each point represents a single water sample. The dashed line indicates −0.3 log_10_, which reflects a linear scale value of 0.5 assigned to non-detect plate counts, or 0 CFU/100 mL (i.e., meeting microbiological standards for “safely managed”). Closed circles indicate rounds after the midline survey round, when dosing was adjusted higher; plus sign symbols indicate rounds before. Each point represents a single water sample. Because systems 1B and 2B share a source and technology installation (with a shared upstream sampling tap), their pretreatment results reflect the same samples. All tap and household samples are unique to their respective systems. (b) Free chlorine (mg/L) across seven monitoring visits from February to November 2020. For each round of sampling, the line connects the specified observed water quality parameter from the tap closest to the chlorinator, the tap farthest from the chlorinator, and one household nearby one of the selected taps. Each point represents a single water sample. The dashed line indicates detectable free chlorine at 0.10 mg/L. Closed circles indicate rounds after the midline survey round, when dosing was adjusted higher; plus signs indicate rounds before.

During seven monitoring visits by NGO staff, the majority of pretreatment samples were contaminated with *E. coli* (Figure 3a). Only three pretreatment samples across all monitoring visits had 0 CFU/100 mL *E. coli*. With the exception of visit round 3, during which two Aquatabs Flo installations were observed to have empty chlorine cartridges, all tap samples had 0 CFU/100 mL *E. coli*, indicating that both technologies were effective over time. Recontamination in the household sample to levels equal to or greater than pretreatment contamination was observed in system 2A. In all other systems, although post-collection recontamination occurred, household stored water quality was better than pretreatment water quality.

During these visits, 81% of taps (closest and farthest) had FCR >0.1 mg/L, although free chlorine declined considerably after household collection and storage (Figure 3b). In system 1B during round 1, observed FCR was higher in the household stored water sample than in either of the taps, although no households reported chlorinating at the household level during surveys. No data on household treatment practices or storage time were collected during monitoring rounds. During round 3 in system 2A, the household sample had 0 CFU/100 mL *E. coli* despite contamination observed at taps.

During frequent FCR monitoring over the 11 months (12 Dec 2019–28 Nov 2019), during which a hired community member measured FCR 1–2 times per week at a single tap per system, an average of 90 (range: 69–97) measurements were collected (Supporting Information Table 2). Across all systems, 74% or more of these tap samples had FCR >0.1 mg/L (74–86% among Aquatabs Flo systems and 90–100% among PurAll 100 systems).

### User Perception and Water Management Behaviors

Household water treatment behaviors remained unchanged throughout the study. At baseline, the majority of households (87%) reported treating their water in the prior 7 days, either by boiling (17%) and/or with a ceramic candle filter (82%). 73% of the samples collected from these ceramic water filters had *E. coli* prior to installation of the chlorinators. At midline and endline, respectively, 79 and 82% of households reported treating their water in the prior 7 days. Across all household visits, most stored drinking water samples were collected from ceramic water filters with taps (77% at baseline, 81% at midline, and 73% at endline). At midline, one respondent reported that their filter was not working.

Over 90% of respondents reported that the taste of water was “good” at all survey rounds (Supporting Information Table 3). However, there was a change in perceived smell, with 87% of respondents identifying either a chlorine or chemical/medicine smell at endline compared to 16% at midline. We increased dosing following the midline survey visit, during which we had observed low dosing. The increased chlorine smell did not translate to an increased perception of drinking water safety. When asked how safe the main drinking water source was for drinking, all respondents across all survey rounds responded either neutrally (“Neither safe nor risky”) or positively (“Quite safe” or “Very safe”). However, the percent of neutral responses increased to 36% at endline, up from 2% at midline and 3% at baseline. Despite the change in smell of water, the study’s community outreach at the start, and multiple visits to the household during which the study was explained, only 67% of respondents said “yes” at endline when asked if the drinking water was treated in any way at the system level. Of these respondents, all correctly said that the treatment included chlorination.

### Observed Costs of Operation and Maintenance

We observed instances of incorrect dosing from both technologies during the study. At the April monitoring visit, chlorine cartridges were empty at two of the installations. During the rainy season, a landslide damaged the intake pipe at the source for system 2A and disrupted service. Subsequently, the flow rate in this system was low, resulting in low dosing. At the endline visit to system 3B, we observed high dosing (4.0 mg/L, the upper limit of detection) at the PurAll 100 installation because a non-return valve downstream of the chlorinator was non-functional; the cause appeared to be built-up sediment. This high dosing resulted in more rapid depletion of the chlorine cartridge. The other PurAll 100 installation had a rapid sand filter installed upstream of the chlorinator; this infrastructure upgrade was planned prior to and installed during the chlorination technology trial and was likely helpful in preventing sediment build up in the chlorinator.

The average installation costs of each device, including all required pipe fittings but excluding both labor and chlorine, were 5290 NPR (46 USD) for Aquatabs Flo and 75675 NPR (662 USD) for PurAll 100 (Table 2). The Aquatabs Flo devices were easily screwed onto to the end of pipes at tanks, while the PurAll 100 devices had more hardware and required cutting the pipe upstream of the tank. Costs will vary for other installations of the same technologies. For example, some installations of Aquatabs Flo in tanks require a second float valve to close a bypass line in tanks that may otherwise fill above the level of the cartridge. Members of the research team supervised initial installation of Aquatabs Flo, which was installed by the NGO staff with assistance from community members, and PurAll 100, which was installed by an NGO technician with assistance from community members. Each installation took less than 2 h, but each PurAll 100 installation required several people to assist. In contrast, the Aquatabs Flo installations required only one or two people. Following installation of the technologies, achieving the correct chlorine dose required multiple visits to each installation by members of the research team, who trained and initially supervised dosing adjustments by the NGO staff.

**Table 2:** Observed Average Installation, Refill, and Monitoring Costs by Technology^b^

	Aquatabs Flo	PurAll 100
completed cartridges	27	5
total volume treated (m^3^)	8318	12,427
average volume (m^3^) treated/cartridge	308	2485
**Installation Costs**		
time required per installation	<1 h	<2 h
hardware cost per installation^a^	5290 NRS (46 USD)	75,675 NRS (662 USD)
**Refill Costs**		
local cost per refill cartridge	3200 NRS (28 USD)	18,000 NRS (157 USD)
average cost chlorine only per m^3^ treated water	0.09 USD	0.06 USD
**Monitoring Costs**		
labor costs for monitoring per m^3^ treated water (as observed in our study)	0.07 USD	0.05 USD

a Including all required pipe fittings and parts and excluding lab costs and chlorine.

b Systems 1B and 2B share a spring source and a single chlorinator installation upstream of their respective reservoir tanks. The total volume value for 1B+2B combines flow meter readings from both tanks.

We calculated the cost of chlorine tablets per cubic meter of treated water to be 0.09 USD for the Aquatabs Flo installations and 0.06 USD for the PurAll 100 installations. In total, there were 27 cartridges completed at Aquatabs Flo installations and 5 at PurAll 100 installations (Supporting Information Table 4). Combining the total volume treated across all installations for each technology over the year, on average, the systems treated 308 m^3^/cartridge (advertized capacity: 180 m^3^/cartridge) for Aquatabs Flo and 2485 m^3^/cartridge (advertized capacity: 2500 m^3^/cartridge) for PurAll 100 (Table 2, Supporting Information Table 4). Differences between advertized and observed cartridge capacities may be explained by periods of low dosing or non-dosing. Since there was no way to quantify partially completed cartridges, only fully completed cartridges are included in chlorine consumption calculations (Table 2).

We calculated labor costs for monitoring, per cubic meter of treated water, to be 0.07 USD for Aquatabs Flo and 0.05 USD for PurAll 100. Since monitoring tasks are equally spread across each system in our study, we allocated costs accordingly. NGO staff and trained community members were all able to install refill cartridges. The two trained local community members conducting regular free chlorine monitoring were each paid for 1.5 days of work per week for a total of 156 person-days per year (52 weeks × 3 person-days/week) to monitor six systems. Each round of monitoring for *E. coli* and total coliforms required 3 days, including travel to the field site, for sample collection and processing by an NGO staff member and for a total of 21 person-days over the year (7 visit rounds × 3 person-days/visit) (Table 3). Technology distributors sent chlorinator supplies to the NGO office, which was located a few hours by car from the study site, via bus from Pokhara and Kathmandu. To avoid supply disruptions during the study, we maintained a supply of refills at the home of one village maintenance worker.

**Table 3:** Observed Chlorine and Labor Cost Calculations over the Entire Study Period (Five Installations, Six Systems)^a^

FCR monitoring	156 person-days/year × 700 NRS/person-day = 109,200 NRS (955 USD)
water quality monitoring	21 person-days/year × 1000 NRS/person-day = 21,000 NRS (184 USD)
total cost of chlorine	Aquatabs Flo: 27 cartridges × 3200 NRS/cartridge = 86,400 NRS (755 USD)
	PurAll 100: 5 cartridges × 18000 NRS/cartridge = 90,000 NRS (787 USD)

a These labor cost calculations reflect average costs across the study installations, but there was variability across sites due to factors such as the distance of tanks from communities and proximity to roads.

Community members voiced concerns about the security of chlorinators, specifically PurAll 100, which was installed just upstream of the reservoir tank. Aquatabs Flo, installed inside the tank at the inlet pipe, was secure because access to tanks required keys that were kept only by village maintenance workers. To protect PurAll 100 installations from vandalism or animals, community members initially covered the devices with branches. Later, the NGO constructed concrete enclosures for the devices; these added an unspecified cost to the installations.

## Discussion

We found that two passive chlorination technologies effectively improved drinking water quality over the course of 1 year in small gravity-fed rural drinking water systems with variable flow rates. At baseline, over 80% of tap samples and over 70% of household stored water samples were contaminated with *E. coli*. One year later, only 7% of taps were positive for *E. coli*, although 29% of household stored water samples still had *E. coli* present. Pretreatment samples collected upstream of the chlorination technologies verified that upstream water quality did not improve over the course of the study. Instead, the improved water quality observed at taps and households was due to effective system-level chlorination.

Passive, system-level chlorination resulted in higher coverage of safely managed water without any behavior change required from, or observed in, individual households. Most households in these communities continued to use ceramic candle filters, which were convenient as covered storage containers but which were not effective at treatment. Since these filters were ineffective on average and because households were transporting their water from taps in various containers and hoses, we expected and observed a decline in water quality between taps and household storage containers.^26^ While 93% of taps had FCR >0.1 mg/L at endline, this was true of only 49% of household stored water samples. Chlorine dissipates and recontamination is a known problem that would reduce FCR, but it is also possible that some household filters contained activated carbon, which removes chlorine, or that the chlorine reacted with metal transport or storage containers, eliminating FCR by the time we measured the stored water. Regardless, household water quality was measurably improved compared to pretreatment water quality, and this risk reduction may result in health benefits even if a protective chlorine residual is not maintained during storage. A study with Aquatabs Flo in urban Dhaka, Bangladesh, found that passive chlorination reduced child diarrhea by nearly a quarter, although free chlorine was detected in only 45% of household stored drinking water samples.^11^

Additional and multi-component water safety interventions would be required to guarantee safe water up to consumption. First, recontamination during transport and storage remained an issue, even with effective system-level chlorination resulting in safe water at the point of collection. The taps in our study were close to households, with an average roundtrip collection time of 6.8 min, but recontamination risks would likely be even greater with longer collection trips. Until households receive reliable and safe water piped into their homes, the promotion of safe transport and storage containers in combination with system-level chlorination is necessary. Second, chlorine is not effective against all pathogens. For example, treating chemical contaminants or protozoa will require that chlorination follow additional treatment steps. However, Orner et al., 2017, found that an in-line, passive chlorinator installed upstream of a tank, similar to our installation, may inactivate most common pathogens at a relatively low FCR because of a sufficiently long chlorine contact time in the distribution system.^12^

Over the course of our study, the cost of labor to monitor and maintain systems was comparable to the cost of chlorine on a per cubic meter of treated water basis. Although maintenance costs vary by setting (e.g., higher in a remote setting with limited road access), they are non-negligible and are crucial for long-term sustainability. Rayner et al. (2016) found that low sustained effectiveness of passive, system-level chlorination in Haiti after 2 years was due to chlorine supply chain issues and the lack of management and maintenance accountability.^27^ In this study in rural Nepal, community water management structures were already in place from prior NGO involvement in water projects, and village maintenance workers were in charge of small repairs. However, when a landslide damaged the intake pipe at the spring source of one system, it remained unfixed for months, and the change in flow rate required chlorinator dosing adjustments. We also observed first-hand the unpredictable supply chain for these imported technologies. Installations for PurAll 100 were delayed because the hardware arrived weeks later than expected. The small, piped water systems in our study communities were effectively treated with passive chlorination, but the NGO was necessary to deliver the chlorine supply and provide regular maintenance support. In other words, our results suggest that the provision of consistently safe water supplies in low-income, small systems such as these requires the support of a service-style delivery model.^28^ We also note that the high upfront capital costs of these systems would make them prohibitively expensive for many communities without NGO or government support with financing.

Our study makes several contributions to the safe water technology literature. First, the year-long, intensive monitoring of the technology installations captures their performance and costs across seasons. We were able to closely track the volume of treated water and refill frequency, to calculate a precise cost of chlorine per cubic meter of treated water, and to roughly estimate the ongoing maintenance costs of both systems. We showed that even when financial costs are no barrier as in these fully funded installations, external organizations may continue to play a key role in sustaining community-based treatment systems over the long term. Second, we found that user perceptions of water quality and safety changed over the course of the year, influenced by both the parent study intervention and increased chlorine dosing. Between midline and endline, we increased chlorine dosing and improved overall water quality, but the smell of chlorine was more noticeable to respondents. Reported taste perceptions did not change. In a previous study in urban Dhaka, respondents were successfully blinded to their assignment to chlorinated water (average 0.37 mg/L at taps) or a placebo, suggesting that respondents either adapted to or did not notice the smell of chlorine.^11^ In our study in rural Nepal, the dosing was higher (average 1.58 mg/L at taps at endline), so the respondent feedback was unsurprising. This change in smell perceptions between midline and endline corresponded with a third of respondents stating at endline that they felt their water was “neither safe nor risky”, increasing from midline (2%) and baseline (3%). This finding does not necessarily indicate that the response to chlorine was negative as no respondents reported that they perceived their water as unsafe across survey rounds. This shift toward more balanced or neutral safety perceptions aligns with the larger REACH-Nepal study, which observed a similar trend among intervention households.^18^ This is likely a result of the intervention successfully increasing awareness about the importance of hygiene and water safety. Overall, user perceptions will change with the introduction of chlorine, although not necessarily for the worse. However, managing user perceptions of chlorine smell may be a more important consideration in settings with alternative non-chlorinated, less safe sources available for household drinking water.

Our study has some limitations. First, the characterization of untreated water quality was based on a single upstream sample for each system at each visit. Water quality is dynamic over time and often declines as it moves through piped systems. However, we observed relatively stable upstream contamination (Figure 3), suggesting that pretreatment samples served as a reasonable proxy for untreated system water quality. Second, our sample size was relatively small, albeit intensively monitored, and the systems had similar infrastructure and source water quality, so our results may have limited generalizability. This is especially true for our cost calculations, although we provide all details of our calculations so that different assumptions for the price of labor and time can be evaluated.

Since the start of this study, additional chlorination technologies have become available,^21^ but limited distribution to and within countries, of both the proprietary technologies and the chlorine tablets themselves, limits the more widespread use of passive chlorination technologies at a low cost. Future research should explore service models that allow communities to easily access chlorine refills. This technology evaluation provides evidence to guide and support the implementation of system-level, passive chlorination technologies, even in low-income, rural communities that are considered challenging settings for successful implementation and maintenance of water treatment infrastructure. Years of research on safe water solutions have established that adoption of household water treatment products is an unrealistic pathway to universal safe drinking water,^29−31^ precisely because it relies on sustained health behavior change. Continuing to rely on household water treatment as the only pathway to low-cost, universal safe water access will leave many behind. In both dense urban and remote rural communities, passive chlorination technologies can improve drinking water quality, without requiring behavior change from individuals in households. Although important questions remain around recontamination risks, reliable supply chains, and service delivery models, these passive treatment approaches have the potential to radically improve how poor households gain access to safe water.

## Data Availability

The data and R scripts to replicate analyses are available at https://osf.io/mrtfb/.

## Supporting Information Available

Additional method details; sample size calculations; lab negative controls; household information; and monthly monitoring data (PDF)

## Notes

Y.S.C. completed this work at the University of California, Berkeley. She is now at the King Center on Global Development at Stanford University.
