# Supplementary material for: Evaluation of System-Level, Passive Chlorination in Gravity-Fed Piped Water Systems in Rural Nepal
Source: Environ Sci Technol. 2022 Sep 20;56(19):13985–95. doi: 10.1021/acs.est.2c03133 (PMC9535811; doi:10.1021/acs.est.2c03133)
Supplement: Supplementary file 1 — es2c03133_si_001.pdf [file es2c03133_si_001.pdf]

Supporting Information for:

An evaluation of system-level, passive chlorination in gravity-fed piped water systems in rural Nepal

Yoshika S. Crider[1,2]*, Sanjeena Sainju[3,4], Rubika Shrestha[4], Guillaume Clair-Caliot[5], Ariane Schertenleib[5], Bal Mukunda Kunwar[4], Madan R. Bhatta[4], Sara J. Marks[5], Isha Ray[1]

1. Energy & Resources Group, University of California, Berkeley, Berkeley, California 94305, United States
2. Division of Epidemiology and Biostatistics, University of California, Berkeley, Berkeley, California 94305, United States
3. Department of Environmental Science and Engineering, Kathmandu University, Dhulikhel 45200, Nepal
4. Helvetas Nepal, Lalitpur 44700, Nepal
5. Eawag, Swiss Federal Institute of Aquatic Science and Technology, Duebendorf 8600, Switzerland

*Corresponding author: ycrider@stanford.edu

Table of Contents

Sample size calculations: ... 3

Lab negative controls: ... 3

Household storage time: ... 3

Monthly monitoring data: ... 3

E. coli contamination in the presence of free chlorine residual: ... 3

SI Table 1. Household baseline characteristics ... 4

SI Table 1 (continued) ... 5

SI Table 2. Regular free chlorine monitoring results ... 5

SI Table 3. User perceptions of water safety and acceptability ... 5

SI Table 4. Observed refill requirements by system ... 6

SI Table 5. Pre-installation average flow rate and chlorine measurements ... 6

SI Figure 1. Total volume of water treated at all systems over time, as recorded from mechanical flow meters. ... 7

SI Figure 2. Risk categories based on World Health Organization guidelines. ... 8

All data are available at https://osf.io/mrtfb/.

Sample size calculations:
To confirm that our feasibility-based sample size was sufficient, we did an *a priori* sample size calculation, clustered at the system level, to determine the minimum number of households per system required to detect a difference in the proportion of households with FCR >0.1 mg/L of 0.60, as compared to an assumed comparison proportion of zero households with chlorine, using an intracluster correlation of 0.10, power equal to 0.80, and alpha equal to 0.05. Only two households per system would be required under this scenario.

Lab negative controls:
40 negative control samples in total were processed during the study. For each negative control we processed 100 mL of sterile water, prepared by boiling tap water with sodium thiosulfate to neutralize any chlorine and then pouring it into a boiled baby bottle. This sterile water was used to moisten compact dry plates during sample processing, and the baby bottle was capped when not immediately in use. 39/40 negative controls had 0 E. coli or total coliforms present. One negative control on Nov 20, 2018, had 1 *E. coli* CFU/100 mL and 2 total coliforms CFU/100 mL. To determine whether the bottle nipple or water had been contaminated, we processed two more negative controls from the same bottle. Both had 0 *E. coli* and total coliforms.

Household storage time:
At baseline, household stored drinking water had been collected an average of 6.8 hours (range: 0.5-24) prior to sampling. At midline, an average of 7.5 hours (range: 1-24). At endline, an average of 7 hours (range: 1-96).

Monthly monitoring data:
During monitoring visits, all negative control lab samples had zero *E. coli* and total coliforms. Some monitoring samples were missing survey entries, resulting in complete *E. coli* data but some missing chlorine measurements. 9 household and tap samples are missing free chlorine measurements, but their *E. coli* and total coliform results are available. Observations with missing data are excluded when calculating summary statistics.

E. coli contamination in the presence of free chlorine residual:
We collected a small number of samples that were positive for *E. coli* even in the presence of free chlorine residual. There are two likely explanations. First, there may have been contamination during sample collection due to failure to practice sterile methods. More likely, there may have been contamination immediately upstream from or at the tap and insufficient chlorine contact time to inactive *E. coli*. Sodium thiosulfate in the Whirlpak Thio-bags immediately neutralizes chlorine residual and would limit chlorine contact time in both cases.

SI Table 1. Household baseline characteristics

	Aquatabs Flo (n=35) Mean (SD)	PurAll 100 (n=36) Mean (SD)	Combined (N=71) Mean (SD)
**Household**			
Respondent age, years	38.5 (14.0)	40.2 (14.4)	39.4 (14.1)
Years lived in community	22.5 (17.1)	29.6 (17.4)	26.1 (17.5)
No formal schooling completed (%)	62.9 (49.0)	63.9 (48.7)	63.4 (48.5)
Completed primary education (%)	17.1 (38.2)	8.3 (28.0)	12.7 (33.5)
Completed secondary education or higher (%)	20.0 (40.6)	27.8 (45.4)	23.9 (43.0)
Number HH members	5.2 (1.8)	4.8 (2.1)	5.0 (1.9)
Number HH members under 5 years	0.6 (0.8)	0.6 (0.8)	0.6 (0.8)
**Assets**			
Owns 1+ radio (%)	42.9 (50.2)	52.8 (50.6)	47.9 (50.3)
Owns 1+ television (%)	2.9 (16.9)	5.6 (23.2)	4.2 (20.3)
Owns 1+ solar panel (%)	100.0 (0.0)	94.4 (23.2)	97.2 (16.7)
Owns 1+ mobile phone (%)	97.1 (16.9)	88.9 (31.9)	93.0 (25.8)
Owns 1+ fridge (%)	2.9 (16.9)	0.0 (0.0)	1.4 (11.9)
Owns 1+ watch (%)	71.4 (45.8)	75.0 (43.9)	73.2 (44.6)
**Main community concerns**			
Healthcare services (%)	60.0 (49.7)	61.1 (49.4)	60.6 (49.2)
Sanitation and hygiene (%)	2.9 (16.9)	41.7 (50.0)	22.5 (42.1)
Transportation and roads (%)	20.0 (40.6)	55.6 (50.4)	38.0 (48.9)
Security and crime (%)	0.0 (0.0)	2.8 (16.7)	1.4 (11.9)
Electricity service (%)	91.4 (28.4)	80.6 (40.1)	85.9 (35.0)
Unemployment (%)	25.7 (44.3)	52.8 (50.6)	39.4 (49.2)
Education (%)	51.4 (50.7)	55.6 (50.4)	53.5 (50.2)
Support for agriculture (%)	22.9 (42.6)	30.6 (46.7)	26.8 (44.6)
Water supply services (%)	11.4 (32.3)	22.2 (42.2)	16.9 (37.7)
**Water access and use**			
Household is involved in community water supply system (%)	48.6 (50.7)	16.7 (37.8)	32.4 (47.1)
Piped water availability, wet season (hours/day)	23.7 (1.5)	14.0 (8.8)	18.8 (8.0)
Piped water availability, dry season (hours/day)	19.7 (6.1)	11.6 (7.5)	15.6 (7.9)
Other domestic water source use, prior 6 months (%)	17.1 (38.2)	-0.0 (0.0)	8.5 (28.0)
Current roundtrip water collection time (minutes)	11.3 (10.0)	5.1 (2.3)	8.2 (7.8)
I collect in containers that I carry (%)	57.1 (50.2)	63.9 (48.7)	60.6 (49.2)
I connect a flexible pipe from the tap to my home (%)	22.9 (42.6)	22.2 (42.2)	22.5 (42.1)
I use both containers and a flexible pipe to my home (%)	20.0 (40.6)	13.9 (35.1)	16.9 (37.7)
Animal water source is piped water supply (%)	54.3 (50.5)	66.7 (47.8)	60.6 (49.2)
Animal water source is another source (%)	14.3 (35.5)	16.7 (37.8)	15.5 (36.4)
Animal water source is both piped water and another source (%)	31.4 (47.1)	16.7 (37.8)	23.9 (43.0)
Number water sources over past year, all purposes	1.6 (0.6)	1.7 (0.5)	1.6 (0.5)

SI Table 1 (continued)

Treatment practices			
Water treatment, prior 7 days	85.7 (35.5)	88.9 (31.9)	87.3 (33.5)
Boiling	31.4 (47.1)	2.8 (16.7)	16.9 (37.7)
Filtration with a tabletop filter	74.3 (44.3)	88.9 (31.9)	81.7 (39.0)
Every day	77.1 (42.6)	72.2 (45.4)	74.6 (43.8)
Half of the time or more	8.6 (28.4)	13.9 (35.1)	11.3 (31.8)

SI Table 2. Regular free chlorine monitoring results

	System ID	N	Proportion tap samples with free chlorine >0.1 mg/L
Aquatabs Flo	1A	92	0.86
	2A	69	0.83
	3A	97	0.74
PurAll 100	1B	96	0.90
	2B	93	0.91
	3B	93	1.00

SI Table 3. User perceptions of water safety and acceptability

	Baseline n=71	Midline N=62	Endline N=55
How is the taste?			
Good	1.00 (0.00)	0.92 (0.27)	0.91 (0.29)
Chlorine	-	0.05 (0.22)	0.09 (0.29)
Chemical	-	0.03 (0.18)	-
How is the smell?			
Good	0.97 (0.17)	0.84 (0.37)	0.13 (0.34)
Chlorine	-	0.11 (0.32)	0.85 (0.36)
Chemical/medicine	-	0.05 (0.22)	0.02 (0.13)
How is the appearance?			
Clear/good	0.87 (0.34)	0.97 (0.18)	1.00 (0.00)
Dirty/cloudy	0.01 (0.12)	0.03 (0.18)	-
How safe do you think your main drinking water source is for drinking?			
Very safe	0.21 (0.41)	0.95 (0.22)	0.27 (0.45)
Quite safe	0.76 (0.43)	0.03 (0.18)	0.36 (0.49)
Neither safe nor risky	0.03 (0.17)	0.02 (0.13)	0.36 (0.49)

All values are proportion (SD)

SI Table 4. Observed refill requirements by system

	Aquatabs Flo			PurAll 100	
System ID	1A	2A	3A	1B+2B	3B
Total volume treated (m^3)	2547	2047	3724	6796	5631
Cartridges completed	8	9	10	2	3
Days elapsed from installation to last recorded refill	335 days	325 days	330 days	292 days	327 days
Volume (m^3) treated per cartridge	318	227	372	3398	1877

SI Table 5. Pre-installation average flow rate and chlorine measurements

Prior to installation of the chlorinator devices, we visited 4 of the systems to collect in-depth flow rate measurements and confirm absence of chlorine. To measure flow rate, we recorded time to fill a 500 mL graduated cylinder; we repeated 3 times and calculated the average. All 9 water samples tested at these points had undetectable (<0.1 mg/L) free and total chlorine.

System	Tank inlet	Tap 1	Tap 2	Tap 3	Tap 4	Tap 5	Tap 6
1	7.0	2.2	0.7	4.4	10.8	6.8	7.9
2	6.5	3.4	5.5	10.0	4.6	4.4	
3	6.3	6.3	7.5	12.9	0.3		
4	6.6	3.5	1.8	2.3	9.5	7.7	

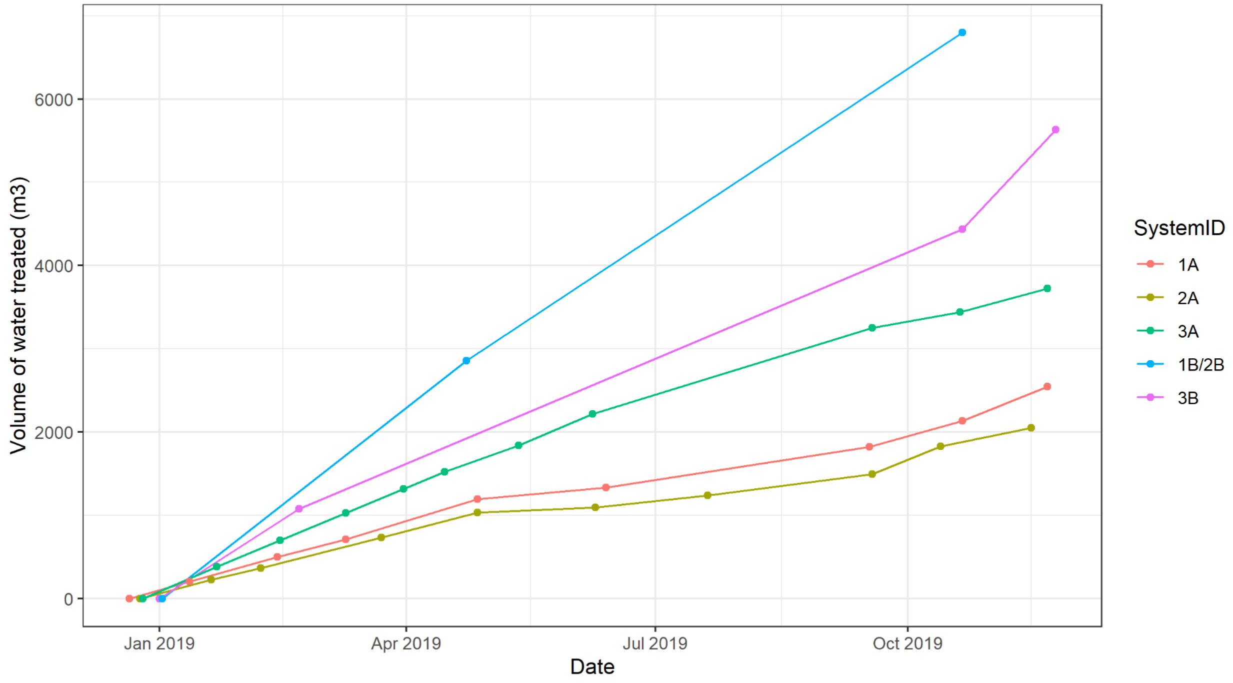

SI Figure 1. Total volume of water treated at all systems over time, as recorded from mechanical flow meters.

Points indicate refills events. Systems 1B and 2B share a spring source and single installation upstream of their respective reservoir tanks. We continued to monitor volume through December 2019, but no additional refills events were recorded after the points shown here.

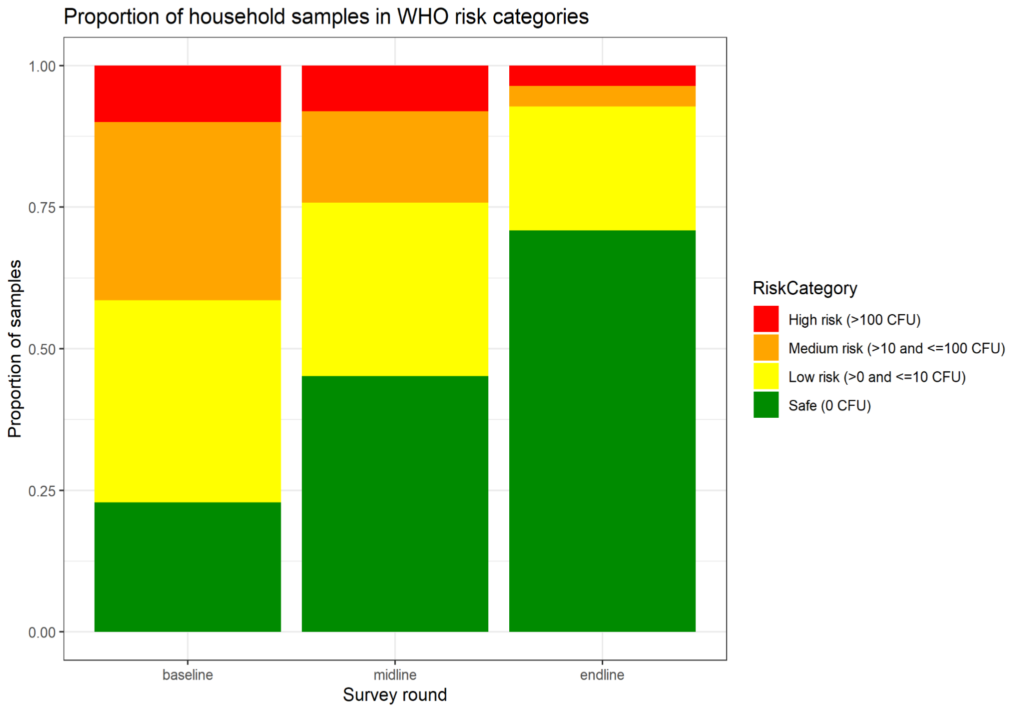

SI Figure 2. Risk categories based on World Health Organization guidelines.

Safe, or meeting guidelines, is 0 CFU *E. coli*/100 mL; low risk is 1-10 CFU; medium risk is 11-100 CFU; high risk is >100 CFU.
